# (*E*)-2-[4-(Dimethyl­amino)­styr­yl]-1-methyl­pyridinium triiodide

**DOI:** 10.1107/S1600536811028753

**Published:** 2011-07-30

**Authors:** Hoong-Kun Fun, Kullapa Chanawanno, Suchada Chantrapromma

**Affiliations:** a X-ray Crystallography Unit, School of Physics, Universiti Sains Malaysia, 11800 USM, Penang, Malaysia; b Crystal Materials Research Unit, Department of Chemistry, Faculty of Science, Prince of Songkla University, Hat-Yai, Songkhla 90112, Thailand

## Abstract

The asymmetric unit of the title compound, C_16_H_19_N_2_^+^·I_3_^−^, contains a (*E*)-2-[4-(dimethyl­amino)­styr­yl)-1-methyl­pyrid­in­ium cation and half each of two triiodide anions. The complete triiodide anions are each generated by inversion symmetry. The planar cation has all of its eighteen non-H atoms situated on a mirror plane. In the crystal, the cations are stacked along the *b* axis by π–π inter­actions with a centroid–centroid distance of 3.5757 (13) Å. The triiodide anions are located between the cations. The crystal structure is further consolidated by short C⋯C [3.322 (9)–3.3952 (19) Å] contacts.

## Related literature

For background to and applications of pyridinium compounds, see: Chanawanno *et al.* (2010 ▶); Fisicaro *et al.* (1990 ▶); Pernak *et al.* (2001 ▶). For related structures, see: Chantrapromma *et al.* (2010 ▶); Zhang *et al.* (2008 ▶). For standard bond lengths, see: Allen *et al.* (1987 ▶). For the stability of the temperature controller used in the data collection, see: Cosier & Glazer (1986 ▶).
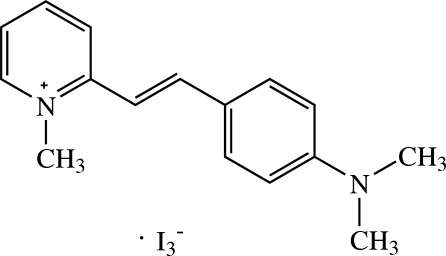


## Experimental

### 

#### Crystal data

C_16_H_19_N_2_^+^·I_3_^−^
*M* *_r_* = 620.03
Monoclinic, 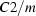

*a* = 19.8760 (3) Å
*b* = 6.6126 (1) Å
*c* = 14.4421 (2) Å
β = 95.107 (1)°
*V* = 1890.62 (5) Å^3^
*Z* = 4
Mo *K*α radiation
μ = 4.96 mm^−1^
*T* = 100 K
0.45 × 0.15 × 0.04 mm

#### Data collection

Bruker APEX DUO CCD area-detector diffractometer
Absorption correction: multi-scan (*SADABS*; Bruker, 2009 ▶) *T*_min_ = 0.212, *T*_max_ = 0.838
16143 measured reflections
2484 independent reflections
2263 reflections with *I* > 2σ(*I*)
*R*_int_ = 0.030

#### Refinement

*R*[*F* ^2^ > 2σ(*F* ^2^)] = 0.030
*wR*(*F* ^2^) = 0.079
*S* = 1.04
2484 reflections
129 parameters
H-atom parameters constrained
Δρ_max_ = 1.74 e Å^−3^
Δρ_min_ = −0.60 e Å^−3^

### 

Data collection: *APEX2* (Bruker, 2009 ▶); cell refinement: *SAINT* (Bruker, 2009 ▶); data reduction: *SAINT*; program(s) used to solve structure: *SHELXTL* (Sheldrick, 2008 ▶); program(s) used to refine structure: *SHELXTL*; molecular graphics: *SHELXTL*; software used to prepare material for publication: *SHELXTL* and *PLATON* (Spek, 2009 ▶).

## Supplementary Material

Crystal structure: contains datablock(s) global, I. DOI: 10.1107/S1600536811028753/sj5182sup1.cif

Structure factors: contains datablock(s) I. DOI: 10.1107/S1600536811028753/sj5182Isup2.hkl

Supplementary material file. DOI: 10.1107/S1600536811028753/sj5182Isup3.cml

Additional supplementary materials:  crystallographic information; 3D view; checkCIF report

## Figures and Tables

**Fig. 1.**
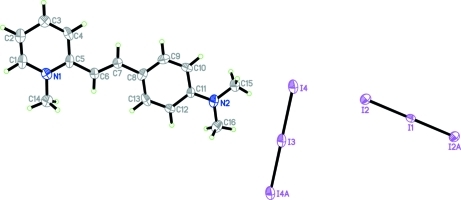
The asymmetric unit of (I) showing 50% probability displacement ellipsoids and the atom-numbering scheme. Atoms I2A and I4A were generated by symmetry codes 1 - x, y, -z and 1 - x, y, 1 - z, respectively whereas one of the three H atoms on each of the three methyl groups are generated by symmetry code x, -y, z.

**Fig. 2.**
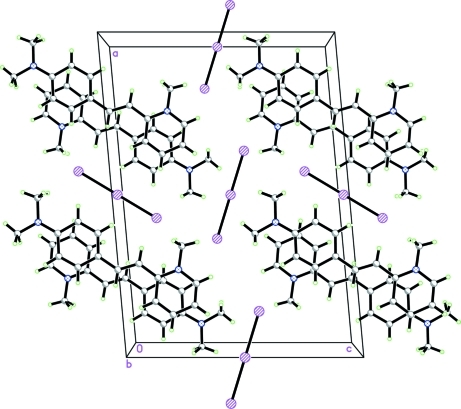
The crystal packing of (I) viewed along the b axis.

## supplementary crystallographic information

### Comment

Pyridinium halide salts, generally possess surface active and interesting antimicrobial properties. They contain reactive functional groups covalently bound to the long hydrophobic chain and can exhibit biological activity (Fisicaro *et al.*, 1990; Chanawanno *et al.*, 2010). It has been proven that one of the factors which control their antimicrobial activity is the presence of anions in the compounds. In the work done by Pernak and coworkers, it was shown that the various anion types can exhibit different antimicrobial activities (Pernak *et al.*, 2001). As our ongoing research is aimed at enhancing the antimicrobial activity of pyridinium salts, we have synthesized pyridinium salts with various anions in order to investigate the relationship between the types of anion and their antimicrobial properties. In the course of this work, the title compound (I) was synthesized and its crystal structure is reported here.

Fig. 1 shows the molecular structure of the title compound (I); the asymmetric unit consists of a C_16_H_19_N_2_^+^ cation and two half-I_3_^-^ anions. The complete molecule of one triiodide anion (I2A) is generated by a crystallographic symmetry centre 1 - *x*, *y*, -*z* whereas the other (I4A) is by 1 - *x*, *y*, 1 - *z*. The cation is 100% planar as all its eighteen non-hydrogen atoms lie on a mirror plane, *x*, 0, *z*. One H atom of each of its three methyl groups at C14, C15 and C16 also lies in the mirror plane. The cation exists in an *E* configuration with respect to the C6═C7 double bond [1.327 (8) Å] and the torsion angles C5–C6–C7–C8 = 180.000 (3)°. The bond lengths (Allen *et al.*, 1987) and angles in (I) are in normal ranges and comparable to those found in related structures (Chantrapromma *et al.*, 2010; Zhang *et al.*, 2008).

In the crystal packing (Fig. 2) the cations are stacked along the *b* axis by π···π interactions with the distances *Cg*_1_···*Cg*_2_ = 3.5757 (13) Å (symmetry codes: 1/2 - *x*, -1/2 + *y*, 2 - *z*; 1/2 - *x*, 1/2 + *y*, 2 - *z*; 1/2 - *x*, -1/2 - *y*, 2 - *z* and 1/2 - *x*, 1/2 - *y*, 2 - *z*); *Cg*_1_ and *Cg*_2_ are the centroids of the C1–C5/N1 and C8–C13, respectively. Triiodide anions are located in the interstitials of the cations. The crystal structure is further consolidated by these π···π interactions and short C···C [3.322 (9)–3.3952 (19)Å] contacts.

### Experimental

The title compound was synthesized by mixing a solution of (*E*)-2-[4-(dimethylamino)styryl]-1-methylpyridinium iodide (Zhang *et al.*, 2008) (0.20 g, 0.55 mmol) in hot methanol (50 ml) and a solution of CuI_2_ (0.17 g, 0.55 mmol) in hot methanol (30 ml). The mixture was stirred for half an hour and then left at room temperature. The title compound was formed as a red solid after 2 days. Orange needle-shaped single crystals suitable for *x*-ray structure determination were obtained by recrystallization from ethanol by slow evaporation of the solvent at ambient temperature over several days, *M*.p. >573 K.

### Refinement

All H atoms were placed in calculated positions with d(C—H) = 0.95 Å, *U*_iso_=1.2*U*_eq_(C) for aromatic and CH and 0.96 Å, *U*_iso_ = 1.2*U*_eq_(C) for CH_3_ atoms. The highest residual electron density peak is located at 0.92 Å from I3 and the deepest hole is located at 0.66 Å from C16.

### Crystal data

**Table:**

C_16_H_19_N_2_^+^·I_3_^−^	*F*(000) = 1152
*M**_r_* = 620.03	*D*_x_ = 2.178 Mg m^−^^3^
Monoclinic, *C*2/*m*	Melting point > 537 K
Hall symbol: -C 2y	Mo *K*α radiation, λ = 0.71073 Å
*a* = 19.8760 (3) Å	Cell parameters from 2484 reflections
*b* = 6.6126 (1) Å	θ = 1.4–28.0°
*c* = 14.4421 (2) Å	µ = 4.96 mm^−^^1^
β = 95.107 (1)°	*T* = 100 K
*V* = 1890.62 (5) Å^3^	Needle, orange
*Z* = 4	0.45 × 0.15 × 0.04 mm

### Data collection

**Table:**

Bruker APEX DUO CCD area-detector diffractometer	2484 independent reflections
Radiation source: sealed tube	2263 reflections with *I* > 2σ(*I*)
graphite	*R*_int_ = 0.030
φ and ω scans	θ_max_ = 28.0°, θ_min_ = 1.4°
Absorption correction: multi-scan (*SADABS*; Bruker, 2009)	*h* = −26→26
*T*_min_ = 0.212, *T*_max_ = 0.838	*k* = −8→8
16143 measured reflections	*l* = −19→18

### Refinement

**Table:**

Refinement on *F*^2^	Primary atom site location: structure-invariant direct methods
Least-squares matrix: full	Secondary atom site location: difference Fourier map
*R*[*F*^2^ > 2σ(*F*^2^)] = 0.030	Hydrogen site location: inferred from neighbouring sites
*wR*(*F*^2^) = 0.079	H-atom parameters constrained
*S* = 1.04	*w* = 1/[σ^2^(*F*_o_^2^) + (0.0387*P*)^2^ + 13.6577*P*] where *P* = (*F*_o_^2^ + 2*F*_c_^2^)/3
2484 reflections	(Δ/σ)_max_ = 0.001
129 parameters	Δρ_max_ = 1.74 e Å^−^^3^
0 restraints	Δρ_min_ = −0.60 e Å^−^^3^

### Special details

**Table:**

Experimental. The crystal was placed in the cold stream of an Oxford Cryosystems Cobra open-flow nitrogen cryostat (Cosier & Glazer, 1986) operating at 100.0 (1) K.
Geometry. All e.s.d.'s (except the e.s.d. in the dihedral angle between two l.s. planes) are estimated using the full covariance matrix. The cell e.s.d.'s are taken into account individually in the estimation of e.s.d.'s in distances, angles and torsion angles; correlations between e.s.d.'s in cell parameters are only used when they are defined by crystal symmetry. An approximate (isotropic) treatment of cell e.s.d.'s is used for estimating e.s.d.'s involving l.s. planes.
Refinement. Refinement of *F*^2^ against ALL reflections. The weighted *R*-factor *wR* and goodness of fit *S* are based on *F*^2^, conventional *R*-factors *R* are based on *F*, with *F* set to zero for negative *F*^2^. The threshold expression of *F*^2^ > σ(*F*^2^) is used only for calculating *R*-factors(gt) *etc*. and is not relevant to the choice of reflections for refinement. *R*-factors based on *F*^2^ are statistically about twice as large as those based on *F*, and *R*- factors based on ALL data will be even larger.

### Fractional atomic coordinates and isotropic or equivalent isotropic displacement parameters (Å^2^)

**Table:**

	*x*	*y*	*z*	*U*_iso_*/*U*_eq_	
I1	0.5000	0.5000	0.0000	0.02351 (12)	
I2	0.424696 (18)	0.5000	0.16345 (3)	0.03339 (11)	
I3	0.5000	0.5000	0.5000	0.03354 (14)	
I4	0.35817 (2)	0.5000	0.42352 (3)	0.03813 (12)	
N1	0.2700 (3)	0.0000	1.2529 (3)	0.0300 (10)	
N2	0.4157 (3)	0.0000	0.6794 (4)	0.0393 (12)	
C1	0.2277 (3)	0.0000	1.3233 (4)	0.0338 (13)	
H1A	0.2472	0.0000	1.3858	0.041*	
C2	0.1609 (3)	0.0000	1.3078 (4)	0.0356 (13)	
H2A	0.1331	0.0000	1.3580	0.043*	
C3	0.1319 (3)	0.0000	1.2140 (4)	0.0329 (12)	
H3A	0.0842	0.0000	1.2007	0.039*	
C4	0.1724 (3)	0.0000	1.1446 (4)	0.0319 (12)	
H4A	0.1530	0.0000	1.0820	0.038*	
C5	0.2453 (3)	0.0000	1.1631 (4)	0.0288 (11)	
C6	0.2883 (3)	0.0000	1.0881 (4)	0.0255 (10)	
H6A	0.3355	0.0000	1.1052	0.031*	
C7	0.2692 (3)	0.0000	0.9977 (4)	0.0275 (11)	
H7A	0.2218	0.0000	0.9817	0.033*	
C8	0.3107 (3)	0.0000	0.9192 (4)	0.0290 (11)	
C9	0.2775 (3)	0.0000	0.8304 (4)	0.0289 (11)	
H9A	0.2295	0.0000	0.8240	0.035*	
C10	0.3105 (3)	0.0000	0.7535 (4)	0.0272 (11)	
H10A	0.2852	0.0000	0.6945	0.033*	
C11	0.3815 (3)	0.0000	0.7579 (4)	0.0232 (10)	
C12	0.4171 (3)	0.0000	0.8475 (4)	0.0272 (11)	
H12A	0.4651	0.0000	0.8536	0.033*	
C13	0.3818 (3)	0.0000	0.9268 (4)	0.0336 (12)	
H13A	0.4059	0.0000	0.9866	0.040*	
C14	0.3424 (3)	0.0000	1.2755 (5)	0.0361 (13)	
H14A	0.3599	0.1166	1.2461	0.043*	
H14B	0.3546	0.0000	1.3414	0.043*	
C15	0.3804 (4)	0.0000	0.5885 (4)	0.0391 (14)	
H15A	0.3516	0.1166	0.5844	0.047*	
H15B	0.4110	0.0000	0.5407	0.047*	
C16	0.4903 (3)	0.0000	0.6862 (5)	0.0460 (16)	
H16A	0.5052	0.1166	0.7219	0.055*	
H16B	0.5073	0.0000	0.6261	0.055*	

### Atomic displacement parameters (Å^2^)

**Table:**

	*U*^11^	*U*^22^	*U*^33^	*U*^12^	*U*^13^	*U*^23^
I1	0.0208 (2)	0.0210 (2)	0.0277 (2)	0.000	−0.00351 (16)	0.000
I2	0.03043 (19)	0.0368 (2)	0.0338 (2)	0.000	0.00747 (14)	0.000
I3	0.0526 (3)	0.0261 (3)	0.0232 (2)	0.000	0.0100 (2)	0.000
I4	0.0541 (3)	0.0367 (2)	0.02383 (19)	0.000	0.00475 (16)	0.000
N1	0.038 (2)	0.023 (2)	0.028 (2)	0.000	−0.0003 (19)	0.000
N2	0.047 (3)	0.044 (3)	0.028 (2)	0.000	0.011 (2)	0.000
C1	0.059 (4)	0.018 (2)	0.026 (3)	0.000	0.014 (2)	0.000
C2	0.054 (4)	0.023 (3)	0.031 (3)	0.000	0.010 (3)	0.000
C3	0.038 (3)	0.023 (3)	0.037 (3)	0.000	0.002 (2)	0.000
C4	0.052 (3)	0.020 (2)	0.022 (2)	0.000	−0.005 (2)	0.000
C5	0.050 (3)	0.015 (2)	0.023 (2)	0.000	0.011 (2)	0.000
C6	0.036 (3)	0.017 (2)	0.024 (2)	0.000	0.004 (2)	0.000
C7	0.033 (3)	0.022 (2)	0.027 (3)	0.000	0.002 (2)	0.000
C8	0.039 (3)	0.017 (2)	0.034 (3)	0.000	0.013 (2)	0.000
C9	0.026 (2)	0.017 (2)	0.044 (3)	0.000	0.005 (2)	0.000
C10	0.027 (2)	0.017 (2)	0.036 (3)	0.000	−0.008 (2)	0.000
C11	0.033 (3)	0.017 (2)	0.021 (2)	0.000	0.0046 (19)	0.000
C12	0.025 (2)	0.025 (3)	0.030 (3)	0.000	−0.003 (2)	0.000
C13	0.052 (3)	0.025 (3)	0.022 (3)	0.000	−0.008 (2)	0.000
C14	0.040 (3)	0.030 (3)	0.038 (3)	0.000	0.005 (2)	0.000
C15	0.052 (4)	0.037 (3)	0.028 (3)	0.000	0.004 (3)	0.000
C16	0.040 (3)	0.056 (4)	0.044 (4)	0.000	0.015 (3)	0.000

### Geometric parameters (Å, °)

**Table:**

I1—I2^i^	2.9054 (4)	C6—H6A	0.9500
I1—I2	2.9054 (4)	C7—C8	1.459 (8)
I3—I4	2.9347 (4)	C7—H7A	0.9500
I3—I4^ii^	2.9347 (4)	C8—C9	1.389 (8)
N1—C5	1.346 (7)	C8—C13	1.407 (9)
N1—C1	1.375 (7)	C9—C10	1.339 (8)
N1—C14	1.448 (8)	C9—H9A	0.9500
N2—C11	1.373 (7)	C10—C11	1.407 (7)
N2—C15	1.433 (8)	C10—H10A	0.9500
N2—C16	1.476 (9)	C11—C12	1.420 (7)
C1—C2	1.327 (9)	C12—C13	1.396 (8)
C1—H1A	0.9500	C12—H12A	0.9500
C2—C3	1.425 (9)	C13—H13A	0.9500
C2—H2A	0.9500	C14—H14A	0.9601
C3—C4	1.341 (9)	C14—H14B	0.9600
C3—H3A	0.9500	C15—H15A	0.9600
C4—C5	1.449 (9)	C15—H15B	0.9598
C4—H4A	0.9500	C16—H16A	0.9600
C5—C6	1.438 (7)	C16—H16B	0.9600
C6—C7	1.327 (8)		
			
I2^i^—I1—I2	180.000 (12)	C8—C7—H7A	115.4
I4—I3—I4^ii^	180.0	C9—C8—C13	117.6 (5)
C5—N1—C1	121.2 (5)	C9—C8—C7	117.5 (5)
C5—N1—C14	119.2 (5)	C13—C8—C7	124.9 (5)
C1—N1—C14	119.6 (5)	C10—C9—C8	122.6 (5)
C11—N2—C15	121.2 (5)	C10—C9—H9A	118.7
C11—N2—C16	120.9 (5)	C8—C9—H9A	118.7
C15—N2—C16	117.9 (5)	C9—C10—C11	121.7 (5)
C2—C1—N1	122.9 (6)	C9—C10—H10A	119.2
C2—C1—H1A	118.6	C11—C10—H10A	119.2
N1—C1—H1A	118.6	N2—C11—C10	122.2 (5)
C1—C2—C3	118.4 (6)	N2—C11—C12	120.6 (5)
C1—C2—H2A	120.8	C10—C11—C12	117.3 (5)
C3—C2—H2A	120.8	C13—C12—C11	120.1 (5)
C4—C3—C2	119.4 (6)	C13—C12—H12A	120.0
C4—C3—H3A	120.3	C11—C12—H12A	120.0
C2—C3—H3A	120.3	C12—C13—C8	120.8 (5)
C3—C4—C5	121.3 (5)	C12—C13—H13A	119.6
C3—C4—H4A	119.4	C8—C13—H13A	119.6
C5—C4—H4A	119.4	N1—C14—H14A	106.9
N1—C5—C6	122.4 (5)	N1—C14—H14B	112.4
N1—C5—C4	116.8 (5)	H14A—C14—H14B	111.7
C6—C5—C4	120.8 (5)	N2—C15—H15A	107.3
C7—C6—C5	127.2 (5)	N2—C15—H15B	111.7
C7—C6—H6A	116.4	H15A—C15—H15B	111.7
C5—C6—H6A	116.4	N2—C16—H16A	107.2
C6—C7—C8	129.3 (5)	N2—C16—H16B	111.9
C6—C7—H7A	115.4	H16A—C16—H16B	111.7
			
C5—N1—C1—C2	0.000 (3)	C6—C7—C8—C13	0.000 (2)
C14—N1—C1—C2	180.000 (2)	C13—C8—C9—C10	0.000 (2)
N1—C1—C2—C3	0.000 (3)	C7—C8—C9—C10	180.000 (2)
C1—C2—C3—C4	0.000 (3)	C8—C9—C10—C11	0.000 (2)
C2—C3—C4—C5	0.000 (3)	C15—N2—C11—C10	0.000 (2)
C1—N1—C5—C6	180.000 (2)	C16—N2—C11—C10	180.000 (1)
C14—N1—C5—C6	0.000 (3)	C15—N2—C11—C12	180.000 (1)
C1—N1—C5—C4	0.000 (3)	C16—N2—C11—C12	0.000 (2)
C14—N1—C5—C4	180.000 (2)	C9—C10—C11—N2	180.000 (1)
C3—C4—C5—N1	0.000 (2)	C9—C10—C11—C12	0.000 (2)
C3—C4—C5—C6	180.000 (2)	N2—C11—C12—C13	180.000 (2)
N1—C5—C6—C7	180.000 (2)	C10—C11—C12—C13	0.000 (2)
C4—C5—C6—C7	0.000 (3)	C11—C12—C13—C8	0.000 (2)
C5—C6—C7—C8	180.000 (2)	C9—C8—C13—C12	0.000 (2)
C6—C7—C8—C9	180.000 (2)	C7—C8—C13—C12	180.000 (2)

Symmetry codes: (i) −*x*+1, −*y*+1, −*z*; (ii) −*x*+1, −*y*+1, −*z*+1.
